# Selective detection of phospholipids using molecularly imprinted fluorescent sensory core-shell particles

**DOI:** 10.1038/s41598-020-66802-3

**Published:** 2020-06-18

**Authors:** Qianjin Li, Sudhirkumar Shinde, Giuliana Grasso, Antonio Caroli, Rahma Abouhany, Michele Lanzillotta, Guoqing Pan, Wei Wan, Knut Rurack, Börje Sellergren

**Affiliations:** 1 Department of Biomedical Sciences, Faculty of Health and Society, Malmö University, SE 205 06 Malmö, Sweden; 2 Chemical and Optical Sensing Division 1.9, Bundesanstalt für Materialforschung und -prüfung (BAM), Richard-Willstätter-Straße 11, 12489 Berlin, Germany; 3 Present Address: Department of Food Science and Engineering, School of Food Science and Pharmaceutical Engineering, Nanjing Normal University, Nanjing, 210023 China; 4 Present Address: Institute for Advanced Materials, School of Materials Science and Engineering, Jiangsu University, Zhenjiang, Jiangsu 212013 China

**Keywords:** Fluorescent probes, Sensors, Nanocomposites, Sensors and biosensors, Fluorescence spectroscopy, Biosensors, Nanoparticles, Biomarkers

## Abstract

Sphingosine-1-phosphate (S1P) is a bioactive sphingo-lipid with a broad range of activities coupled to its role in G-protein coupled receptor signalling. Monitoring of both intra and extra cellular levels of this lipid is challenging due to its low abundance and lack of robust affinity assays or sensors. We here report on fluorescent sensory core-shell molecularly imprinted polymer (MIP) particles responsive to near physiologically relevant levels of S1P and the S1P receptor modulator fingolimod phosphate (FP) in spiked human serum samples. Imprinting was achieved using the tetrabutylammonium (TBA) salt of FP or phosphatidic acid (DPPA·Na) as templates in combination with a polymerizable nitrobenzoxadiazole (NBD)-urea monomer with the dual role of capturing the phospho-anion and signalling its presence. The monomers were grafted from ca 300 nm RAFT-modified silica core particles using ethyleneglycol dimethacrylate (EGDMA) as crosslinker resulting in 10–20 nm thick shells displaying selective fluorescence response to the targeted lipids S1P and DPPA in aqueous buffered media. Potential use of the sensory particles for monitoring S1P in serum was demonstrated on spiked serum samples, proving a linear range of 18–60 µM and a detection limit of 5.6 µM, a value in the same range as the plasma concentration of the biomarker.

## Introduction

Beyond their role to maintain the integrity of living cells, several lipids are indispensable factors in cellular signalling^1–4^. Many diseases have been linked with individual lipid species or related metabolic disorders^5^. In view of the dynamic nature of these processes the time and space resolved quantification of these molecules is crucial for both fundamental understanding and for developing improved diagnostic tests^6–9^. Robust means of real-time lipid quantification *in situ* are still to be realized. This warrants the development of affinity probes e.g. immune-, receptor or aptamer-based sensors^10,11^, capable of continuously reporting the lipid levels in biofluids.

Such probes would be particularly beneficial for monitoring sphingosine-1-phosphate (S1P) in blood or in living cells, a signalling lipid rapidly emerging as a biomarker for a variety of conditions comprising among others cancer^4^, multiple sclerosis^12^, cardiovascular disease^13^ and Alzheimer´s disease^14^. Of equal urgency are probes for fingolimod (FTY720), a sphingosine analog and S1P-receptor modulator  used in the treatment of multiple sclerosis^15,16^.

Addressing the robustness issue of biological receptors, lipid recognition elements in the form of macrocyclic hosts have been reported^17–20^. However, these typically lack the required target selectivity and are often synthetically challenging to make.

Molecular imprinting offers a possible solution to these problems^21–30^. Polymers (molecularly imprinted polymers = MIPs) are prepared in presence of a template, structurally resembling or identical to the target that the polymers are designed to bind. Following this step, the template is removed, leaving behind a binding site complementary to the target molecule. Like antibodies, such receptors can be used for affinity-based separations, assays or sensors for the target analytes. MIPs featuring optically responsive properties offer a particularly attractive means of target detection^24–29^.

In order to adapt this approach for an S1P-probe, we set out the following design criteria:

1. Lacking effective template recycling rules out the use of expensive targets as templates. Most phospholipids belong to this category which explains why only few examples of MIPs targeting phospholipids have been reported^21,22,30^. We reasoned that an S1P complement can be constructed based on templating of the readily available S1P receptor modulator fingolimod phosphate (Fig. 1). This zwitterionic drug antagonizes the receptor by a similar binding mechanism as S1P^16^.

**Figure 1:**
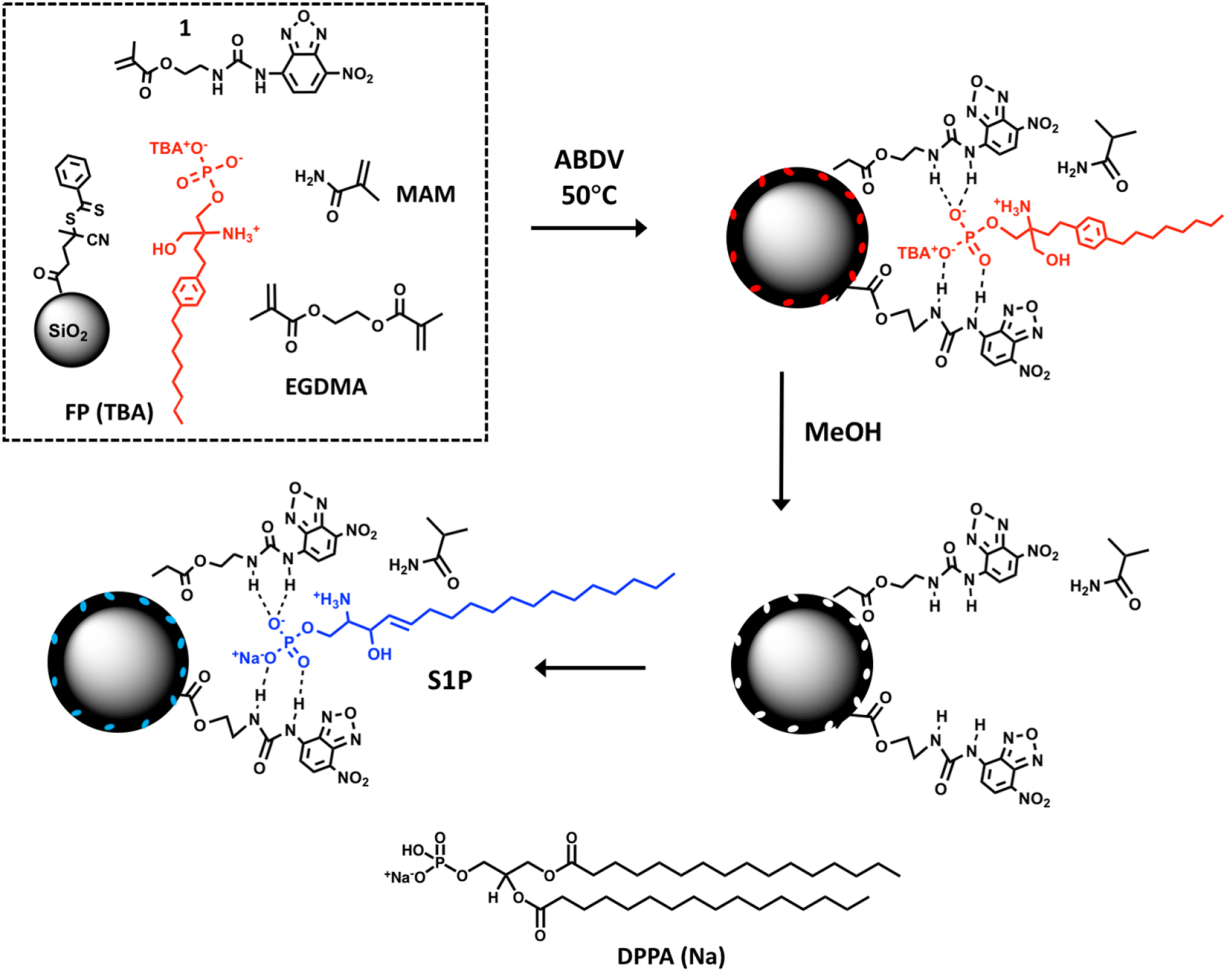
Procedure for RAFT-mediated grafting of a FP(TBA) imprinted shell on silica core particles based on hydrogen bond stabilization using NBD-urea monomer (**1**). After template removal the polymer is ready to accommodate S1P leading to guest induced fluorescence modulation. The protonation state of FP is based on the proposed charge state of S1P bound to its receptor^31^. MAM: methacrylamide; EGDMA: ethyleneglycol dimethacrylate. Figure created by authors using Chemdraw Professional v. 17.1 (URL: https://www.perkinelmer.com/se/category/chemdraw) and MS Power Point v. 16.35 (URL: https://www.microsoft.com/).
2. The amphiphilic nature of the template/target requires an amphiphilic host capable of accommodating the polar head group and the hydrophobic chain. In our previous efforts towards a receptor for the lipid A motif of endotoxin, the phosphomonoester head group could be effectively targeted based on cationic bis-imidazolium or neutral urea-based anion host monomers in a hydrophobic poly-methacrylate scaffold^30^.
3. Real time lipid quantification in live cells is complicated by the fact that lipids are largely associated with proteins or cell membranes. Probes compatible with denaturing media are therefore required. The MIP should hence report the presence of a guest with a short response time in both aqueous and non-aqueous media. Preparation of submicron-sized core/shell particles incorporating fluorescent reporter monomers such as ureas with appended nitrobenzoxadiazole (NBD) fluorophore groups has proven to be a fruitful approach for generating target specific and polymerizable fluorescent probes featuring organic solvent compatibility combined with short response times^25,26,28^.

Based on the above design criteria, we here report on the synthesis and characterization of fluorescent particle probes for the phosphomonoester lipids S1P, phosphatidic acid and the S1P receptor modulator fingolimod-phosphate (FP)^15^.

## Results and Discussion

Use of equimolar amounts of NBD-urea monomer **1** and the monosodium or TBA salt of FP or DPPA in combination with RAFT mediated grafting (Fig. 1) we anticipated would lead to lipid recognitive surface sites with guest-sensitive optical response.

According to conventional supramolecular chemistry reasoning, urea-based oxyanion probes exploit the directional hydrogen bonds offered by the two-fold hydrogen bond donor motif of the host and the Y-shaped oxygen acceptor motif of a carboxylate guest^32^. A key rationale in the design of such hosts is to tune the donor acidity to maximize complex formation while minimizing deprotonation. The latter is usually undesired since it either leads to reprotonation of the oxyanion guest by the host, potentially leading to attraction between anionic host and the initial oxyanion counterion, or even to electrostatic repulsion between ureate host and deprotonated oxyanion guest. Ureate formation is commonly visible in the titration spectra of ureas and oxyanions by a strongly red-shifted absorption band (often by ca. 100 nm)^32^.

**Figure 2:**
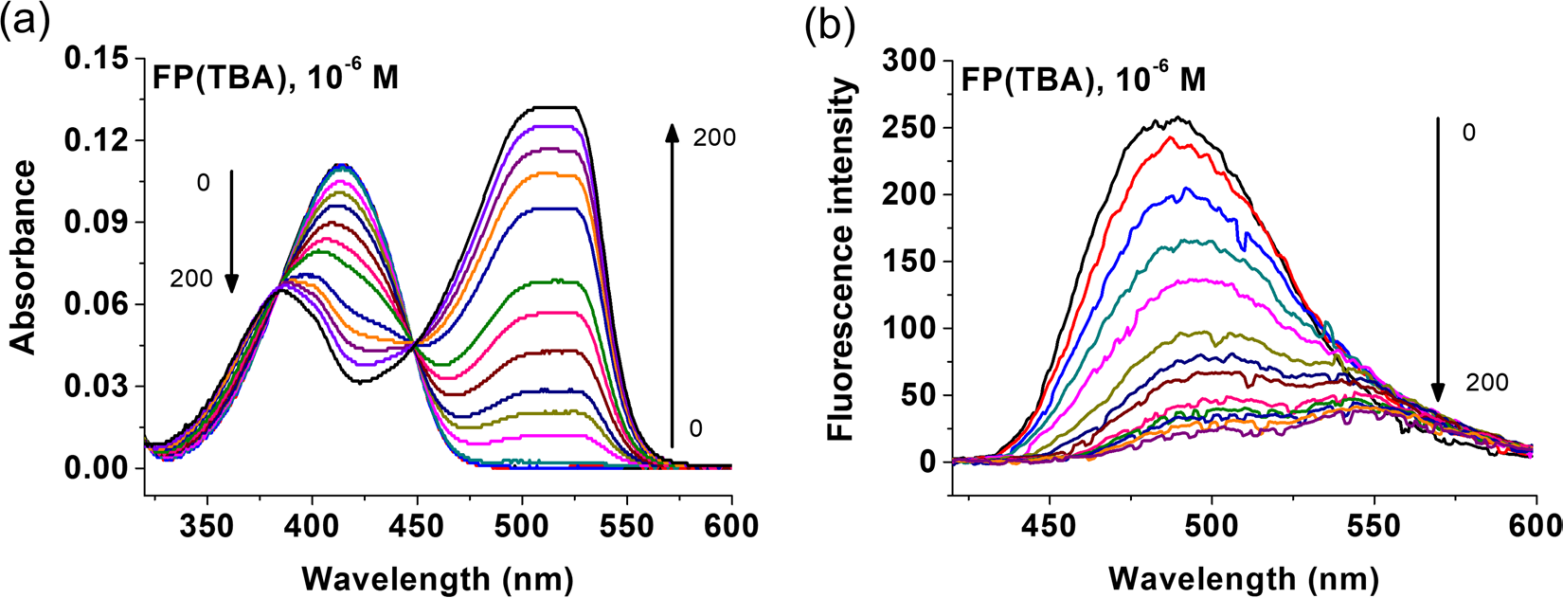
Absorption (**a**) and fluorescence (**b**) spectra of **1** (c = 10 μM in CHCl_3_) in absence (black) and presence of FP(TBA). Excitation wavelength: 410 nm. Figure created by authors using Origin v. 9 (URL: https://www.originlab.com).

Figure 2 shows the spectroscopic titration of **1** with the mono-tetrabutylammonium (TBA) salt of FP in chloroform. Both absorption and fluorescence spectra reveal the appearance of red-shifted bands immediately upon guest addition. A similar behaviour was observed for titrations carried out in methanol or using the divalent salt FP (2TBA). This shows that for the title system indeed deprotonation is a dominant process resulting in an anionic host and a neutral guest. Although deprotonation is commonly detrimental to achieving directional imprinting, the case of FP is somewhat different. First, it is an amphiphile with two molecular subunits of very different hydrophilicity. Second, it carries an amino group in close proximity to the phosphate head group, potentially creating a zwitterionic species. If another proton is abstracted by forming the TBA salt of FP and using this species as template for imprinting, electrostatic forces prevail and an ensemble of **1**^−^ / H_3_N^+^–FP–HPO_4_^−^ / TBA^+^, with TBA^+^ being more lipophilic than **1**^−^, will orient in a way that the ionic head of FP is directed toward **1**^–^. We thus moved on and imprinted FP(TBA) with **1** according to our previously reported procedure^25,28^.

**Figure 3:**
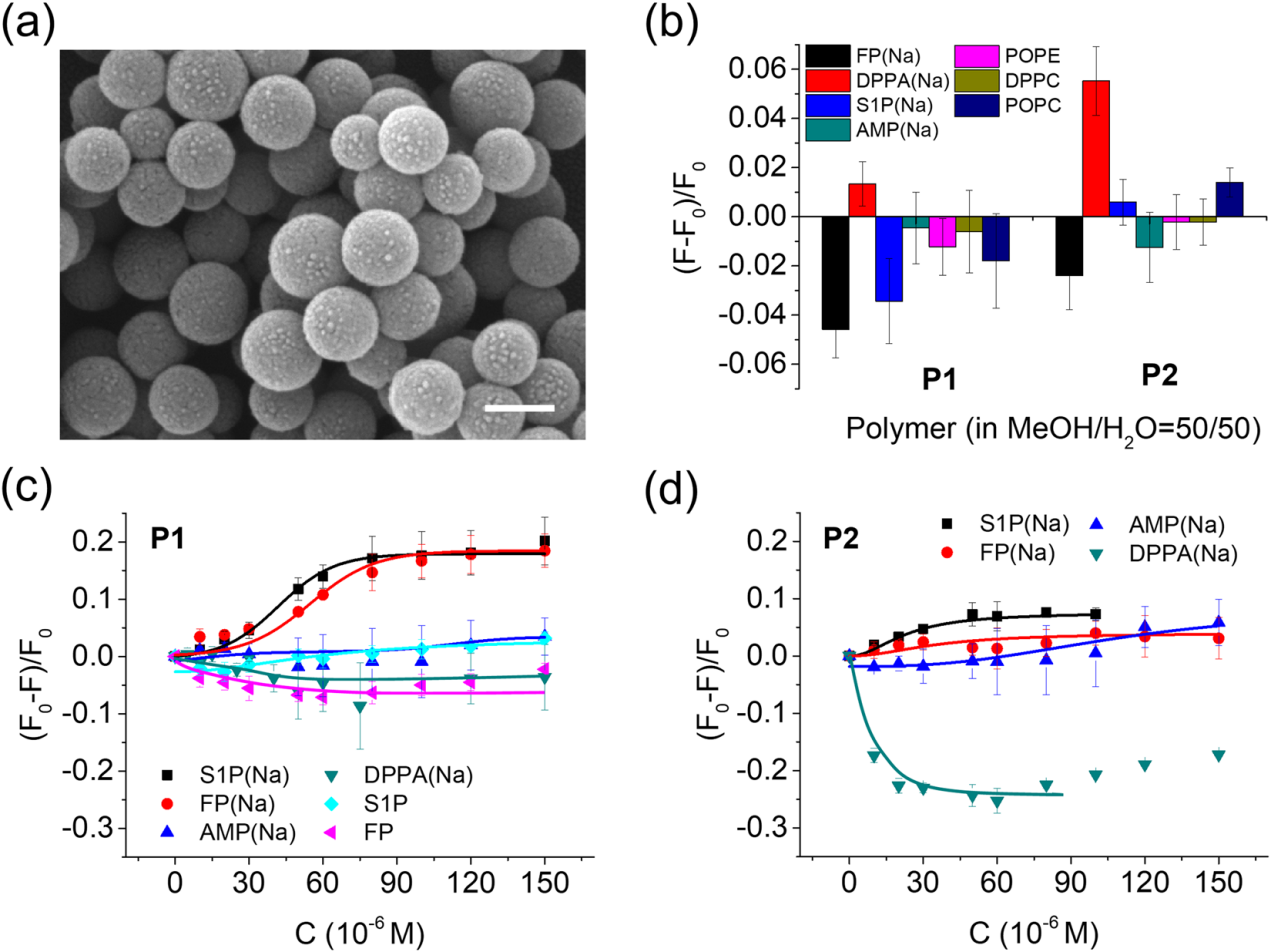
(**a**) SEM image of **P1** (scale bar = 200 nm). (**b**) Fluorescence signal response to the indicated compounds (20 µM) in methanol/water = 50/50 (**c,d**) Fluorescence titration curves of test compounds added to polymers **P1** (**c**) and **P2** (**d**). The lines are drawn as a guide for the eye. Polymer concentration: 1 mg/mL. Figure created by authors using Origin v. 9 (URL: https://www.originlab.com).

For imprinting, the monomers were grafted from ca 300 nm modified silica core particles by reversible addition fragmentation chain transfer (RAFT) polymerization^25,26,28^, with a molar feed ratio of FP(TBA):**1**:MAM:EGDMA equal to 1:2:40:200 and with the beads dispersed in chloroform. We used 2 equivalents of **1** to potentially achieve better stabilization of zwitterionic FP(TBA) (Fig. 1). In addition to the FP imprinted polymer **P1**, a nonimprinted control polymer (**P**_**N**_**1)** and a polymer prepared using DPPA(Na) as template (**P2**) were prepared. After washing and template removal by repeated solvent extractions the beads were characterized by FTIR, TGA, DLS, TEM and SEM (Table S1, Figs. S1–S3). The FTIR spectra of the core shell beads (Fig. S1a) display the characteristic carbonyl stretching of the polymer matrix at ca 1740 cm^−1^ and the siloxane vibration of the silica core at ca 1050 cm^−1^ .  The ratio of their intensities scale with the density of grafted polymer indicating a somewhat higher grafting yield for **P2**. TEM and SEM images confirmed the grafting (Figs. 3a, S1 and S2) and core shell architecture (Fig. S1c) with shells appearing brighter due to their lower electron density. The shell thickness was estimated to 10–20 nm. Both the DLS results and SEM images indicate particle aggregation appearing more severe for **P2** featuring the higher grafting density.

The particles were then tested for their affinity and fluorescence response towards the templates (Figs. S4 and S5). Addition of FP(TBA) to **P1** and **P**_**N**_**1** in methanol/water: 95/5 (v/v) resulted in uptake and fluorescence quenching with a relatively small difference between P1 and the nonimprinted reference P_N_1. To investigate the effect of added water, the test was repeated for the sodium salt of the template FP(Na) in a series of methanol/water mixtures (Figs. S6–S8). Increasing the water content led to a pronounced increase in the adsorbed amount of template (Fig. S6), an effect presumably driven by increased hydrophobic interactions. This was accompanied by a transition from binding induced quenching to enhancement of fluorescence (Fig. S7). This contrasts with the behavior of **P2** vis-à-vis DPPA(Na) which in all solvent systems displayed enhancement effects. Given the different hydrophobicities of these templates as reflected in their LogP values (Table S2) we postulate this effect to depend on the microenvironment induced by template uptake^33^, DPPA resulting in a lower polarity and hence less urea deprotonation. The rapid binding kinetics (Fig. S8) with equilibria reached within ca 10 min agreed with results in our previous report^25^.

In view of the contrasting fluorescence response of FP and DPPA we decided to focus a more detailed investigation of the polymers in methanol/water: 50/50 (v/v) which corresponds to the solvent system causing the largest response difference. A sensor operating in this solvent system would have the additional benefit of reducing matrix effects and masking of the target lipids due to protein binding. Fig. 3b–d show the polymers fluorescence response to a series of phospholipids including S1P and the non-related cofactor AMP.

In spite of the rather shallow titration curve (Fig. 3c), **P1** displayed a significant selectivity for FP(Na) and the single chain lipid S1P(Na) as manifested in enhanced quenching upon addition of these molecules and only minor changes when adding AMP(Na), DPPA(Na) or the zwitterionic targets. In contrast, **P2** displayed a notable signal only in presence of DPPA(Na) (Fig. 3d) but this time in the form of enhanced fluorescence. Significantly smaller changes were observed upon addition of the control lipids or AMP(Na). The response was more sensitive reflecting an affinity of K_d_ < 10 µM for DPPA(Na). This pronounced effect may be due to the enhanced hydrophobicity of this target and/or the lack of the zwitterionic character.

We next decided to challenge the polymers with an extended range of lipids comprising both phospho-monoesters and diesters (Fig. 3b). As expected, both polymers showed the strongest responses (**P1** = quenching; **P2** = enhancement) for their complementary guests and only weak responses for the phosphodiesters and AMP. This strongly indicates that the fluorescence response involves docking of the guests to templated sites.

**Figure 4:**
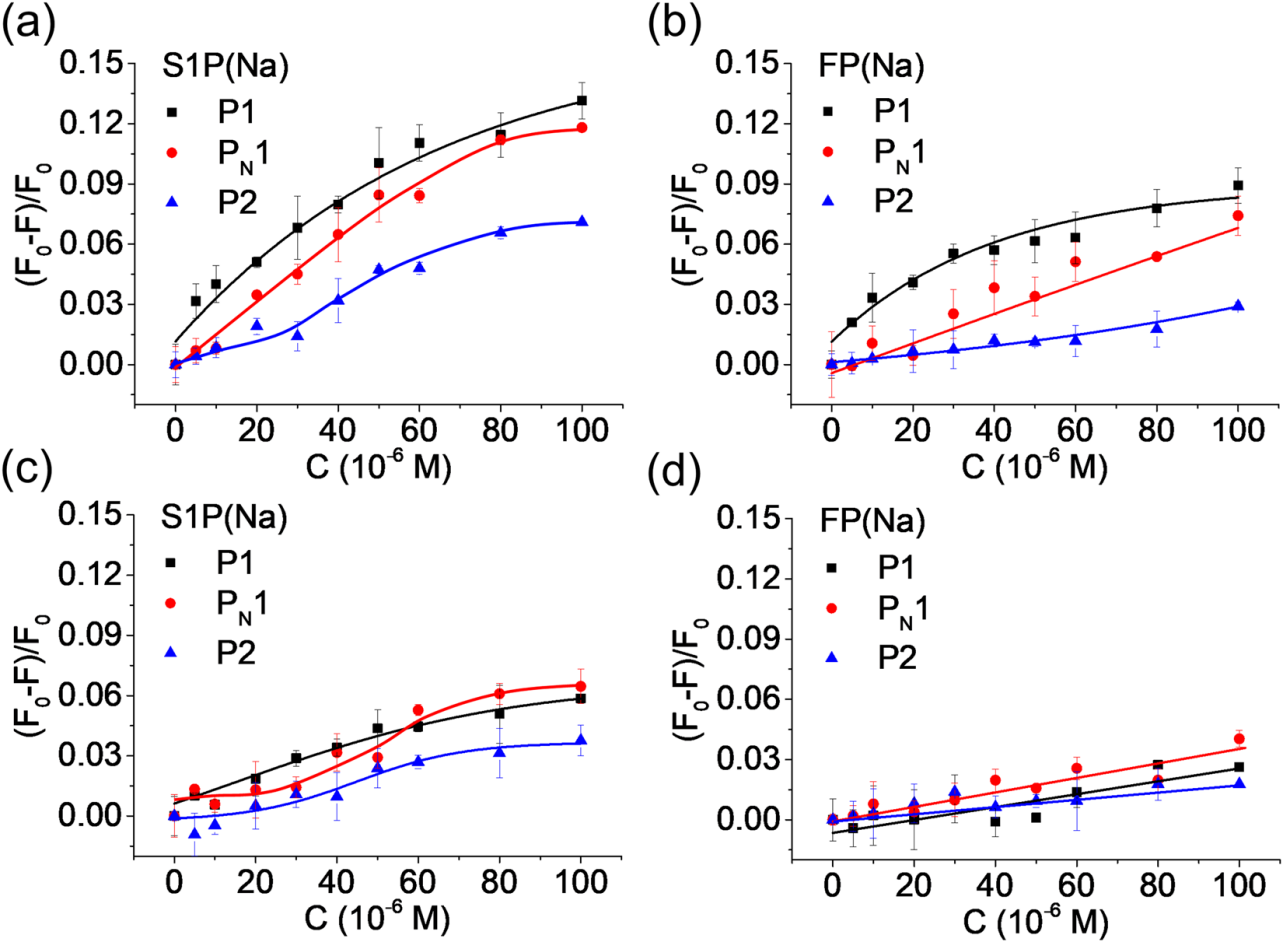
Dose-response behaviours of the polymers to the indicated lipids in diluted human serum (25-times dilution in methanol/water:1/1) in the absence (**a,b**), and in the presence of FP(Na) (**c**, 100 μM) and S1P(Na) (**d**, 100 μM). The lines are drawn a a guide for the eye. Polymer concentration: 1 mg/mL. Figure created by authors using Origin v. 9 (URL: https://www.originlab.com).

Competitive binding experiments further supported this explanation. Figure S9 shows the fluorescence response of the three polymers when titrated with FP(Na), S1P(Na) and DPPA(Na) in presence of potential inhibitors. Whereas addition of structurally unrelated molecules such as in Fig. S9a,c did not affect the selective sensor response, addition of the template FP selectively suppressed the S1P signal of its polymer complement  **P1**(Fig. S9b). Similar effects were observed when titrating the polymers in dilute human serum (Fig. 4). Hence, S1P(Na) effectively suppressed the **P1**-response to FP(Na) and conversely FP(Na) suppressed the signal of S1P(Na).

To test whether the probes could detect S1P in spiked 4x diluted human serum we recorded dose/response curves of **P1** to the targets S1P(Na) and FP(Na) (Fig. S10). Interestingly, these curves displayed larger initial slopes and regression coefficients. Assuming the response to reflect binding, curve fitting to a 1:1 binding model gave a dissociation constant K_d_ = 17 µM for S1P and 18 µM for FP (Table 1). With an estimated linear range of 18–60 µM and an LoD of ca 6 µM we decided to measure samples spiked with high concentrations of S1P, exceeding physiologically relevant values.

**Table 1:** Accuracy evaluation using spiked serum matrix as unknown sample.

Target	Linear range (µM)	Expected conc. (μM)	Observed conc. (μM)	LoD (µM)	K_d_ (µM)
S1P(Na)	18–60	20	25 ± 3	5.6	17 ± 1.6
FP(Na)	18–80	20	25 ± 8	4.0	18 ± 1.7

As seen in Table 1, the sensor could determine the concentration of S1P and FP in the samples with a systematic positive error of ca 25%.

## Conclusions

In summary, we have used our previously reported fluorescent host monomer combined with a rational selection of template, polymer scaffold and particle architecture to prepare fluorescent phospho-lipid probes for the signalling lipid S1P and the S1P-receptor modulator  FP. The probes display short response times and pronounced lipid recognition for measurements in dilute human serum. In view of the LoD exceeding ca 5x the physiologically relevant concentration (ca 1 µM) further work is required to translate this concept into a practically useful system. Nevertheless, we consider this work as the first step towards a general sensor platform for phospholipids.

## Methods

### Preparation of core-shell particles

RAFT modified silica nanoparticles (SiNP-RAFT, see Supporting Information) (150 mg) were suspended in anhydrous chloroform (20 mL) by ultra-sonication. Then, the template, FP(TBA) (**P1**) or DPPA (Na) (**P2**) (4.8 mg or 5.1 mg, 7.6 µmol), fluorescent functional monomer 1 (5.1 mg, 15.2 µmol), co-functional monomer MAM (26 mg, 306 µmol) and crosslinker EGDMA (297.3 mg, 1.5 mmol) were added into the suspension. The mixture was subjected to sonication for 30 min followed by purging with nitrogen during 20 min. ABDV (8.7 mg, 35.0 μmol) was then added and the suspension again purged for 5 min with nitrogen. The vial was sealed with silicone insulating tape and stirred on a heated shaker (50 °C, 480 rpm) for 23 h. The synthesized core-shell particles were collected by centrifugation at 10000 rpm, washed with acetonitrile/chloroform = 70/30 (v/v) three times (each 40 mL) and methanol successively, and dried under vacuum at room temperature overnight. A non-imprinted polymer (**P**_**N**_**1**) was synthesized identically but in the absence of template. The surface coverage of the SiO_2_ nanoparticles with APTES and CPDB and the shell thickness of the core-shell particles were assessed with thermogravimetric analysis (TGA). The size and core/shell structure of the nanoparticles were verified by transmission electron microscopy (TEM).

### Binding tests

Binding tests were carried out as follows. Core-shell particles (1 mg or 10 mg) were suspended in a 1 mL solution of the analyte (100 µM) in a centrifuge tube. After 4 h incubation at room temperature by gentle shaking, the suspension was centrifuged at 10000 rpm for 10 min. The supernatant was transferred to HPLC vial for measurement of the unbound analyte by reversed phase HPLC analysis^9^.

### Kinetic fluorescence measurement

A suspension of core-shell particles (1 mg mL^−1^) was prepared in methanol/water at different volume ratios (2 mL). The fluorescence emission intensity (F_0_) of the suspension was measured at 512 nm (λ_ex_ = 440 nm). After addition of test molecules, the suspension was stirred for 30 seconds and the fluorescence emission intensity (F) again measured. The procedure was repeated at different time intervals as indicated in Fig. S8.

### Assay protocol for sensing

A 2 mL suspension of core-shell particles (1 mg mL^−1^) was prepared using solvents as specified. The fluorescence emission spectrum of the suspension was measured (λ_ex_ = 440 nm). The suspension was then titrated with 1.0 mM solution of the respective lipids. After each addition, the suspension was allowed to equilibrate under constant stirring for 10 min before recording the fluorescence spectra.

### Detection of S1P and FP in human serum

Frozen human serum was first thawed at room temperature. Equivalent volumes of the thawed serum and methanol were thereafter mixed and shaken for 30 min followed by centrifugation at a speed of 10000 rpm for 15 min. The supernatant was further diluted by methanol/water = 1/1 (v/v) in presence or absence of the core-shell particles. This solution was spiked with known concentrations of FP (Na) or S1P (Na) and the fluorescence emission measured as described above. To test the accuracy of the sensor, human serum spiked with targets (FP(Na) or S1P(Na)) was mixed with 1 mL methanol for 30 min. After centrifugation, 1 mL supernatant was mixed with 1 mL of a suspension (2 mg/mL) of the core-shell particles in methanol/water = 1/1 (v/v). The suspension was stirred for 10 min followed by measurement of the fluorescence intensity at 512 nm (λ_ex_ = 440 nm). The results correspond to averages of three independent experiments. Assuming a linear correlation between fluorescence signal and adsorbed amount of analyte we used equilibrium binding analysis to estimate the dissociation constant, K_d_ for the analyte-particle interaction. The response curves in Fig. S10 were hence fitted to the Langmuir single site model using Graphpad Prism v7.0. The limit of detection (LoD) was estimated as the concentration producing a signal corresponding to a minimum of three times the standard deviation (SD) of the blank signal.

## Supplementary information

Supplementary Information.
