# Supplementary material for: Selective detection of phospholipids using molecularly imprinted fluorescent sensory core-shell particles
Source: Sci Rep. 2020 Jun 18;10:9924. doi: 10.1038/s41598-020-66802-3 (PMC7303128; doi:10.1038/s41598-020-66802-3)
Supplement: Supplementary file 1 — Supplementary Information. [file 41598_2020_66802_MOESM1_ESM.pdf]

Supporting Information

Selective detection of phospholipids using molecularly imprinted fluorescent sensory core-shell particles

Qianjin Li,[a,c] Sudhirkumar Shinde,[a] Giuliana Grasso,[a] Antonio Caroli,[a] Rahma Abouhany,[a] Michele Lanzillotta,[a] Guoqing Pan,[a,d] Wei Wan,[b] Knut Rurack,[b] and Börje Sellergren*[a]

[a.] Department of Biomedical Sciences, Faculty of Health and Society, Malmö University, SE 205 06 Malmö, Sweden. E-mail: borje.sellergren@mau.se (B. Sellergren)

[b.] Chemical and Optical Sensing Division 1.9, Bundesanstalt für Materialforschung und -prüfung (BAM), Richard-Willstätter-Straße 11, 12489 Berlin, Germany.

[c.] Current address: Department of Food Science and Engineering, School of Food Science and Pharmaceutical Engineering, Nanjing Normal University, Nanjing 210023, China

[d.] Current address: Institute for Advanced Materials, School of Materials Science and Engineering, Jiangsu University, Zhenjiang, Jiangsu, 212013, China Address here.

Table of contents

1.1. Materials and instruments.. 2
1.2. Preparation of RAFT modified silica nanoparticle (SiNP-RAFT)............................. 3
1.3. References 4
2. Figures and tables... 5

1. Experimental section

1.1. Materials and instruments

Fingolimod phosphate (FP) was supplied by Novartis Institutes for BioMedical Research (Switzerland). Sphingosine-1-phosphate (d18:1) (S1P), 1-palmitoyl-2-oleoyl-sn-glycero-3-phosphoethanolamine (16:0-18:1) (POPE), 1,2-dipalmitoyl-sn-glycero-3-phosphocholine (16:0) (DPPC) and 1-palmitoyl-2-oleoyl-sn-glycero-3-phosphocholine (16:0-18:1) (POPC) were from Avanti Polar Lipids company (USA). Tetraethyl orthosilicate (TEOS), 3-aminopropyltriethoxysilane (APTES), 4-cyano-4-(thiobenzoylthio)pentanoic acid) (CPDB), ethylene glycol dimethacrylate (EGDMA), triethylamine (TEA), tetrabutylammonium hydroxide solution (TBA-OH, 1.0 M in methanol), ethyl chloroformate, chloroform (anhydrous), methacrylamide (MAM), adenosine 5'-monophosphate (AMP) and 1,2-dipalmitoyl-sn-glycero-3-phosphate monosodium salt (16:0) (DPPA(Na)) were obtained from Sigma Aldrich (Steinheim, Germany). Methanol came from Acros Organics (Geel, Belgium). Acetonitrile was obtained from Merck (Darmstadt, Germany). EGDMA was passed through a column of activated basic alumina to remove inhibitor and stored at -20 °C before polymerization. The fluorescent functional monomer 2-(-3-(4-nitrobenzo[c][1,2,5]oxadiazo-7-yl)ureido)ethylmethacrylate (1) was synthesized according to our previously published protocol.[1] Milli-Q water was obtained with a Milli-Q® ultrapure water purification system (Millipore Synthesis A10). Human serum (human male AB plasma, USA origin, sterile-filtered) was bought from Sigma Aldrich (Steinheim, Germany).

TBA salts of the phospholipids (e.g. FP(TBA)) were prepared by mixing an equimolar amount of the lipid and TBA-OH in methanol overnight, followed by drying under vacuum at room temperature. Correct stoichiometry was verified by ^{1}H-NMR.

Fluorescence spectra were recorded on a LS-50B spectrofluorometer (Perkin Elmer, UK) equipped with a magnetic stirrer. The HPLC measurements were carried out on an Agilent 1100 instrument equipped with a UV-DAD detector and an autosampler. FTIR

(ATR) spectra were recorded on a Nicolet 6700 instrument with a SmartITR accessory, a standard KBr beamsplitter, a spectral range of 5000-400 cm^{-1}, and a resolution of 4 cm^{-1}. All spectra were processed and analysed using the OMNIC 8 software. The polymerizations were carried out using a KS130 basic orbital shaker (IKA Stauffen, Germany) equipped with a dry block heater. SEM images were recorded on a S4800 scanning electron microscope (Hitachi, Japan). TEM images were recorded on a Tecnai G2 transmission electron microscope (FEI, USA). Particle sizes were measured by a Zeta Potential/Particle Sizer NICOMP™ 380 ZLS instrument with ZPW388 software from Particle Sizing Systems (USA). The obtained particles (1mg) were dispersed in methanol to a concentration ～0.1 mg mL^{-1} and sonicated for 10 min, then analysed by DLS at 25°C.

1.2. Preparation of RAFT modified silica nanoparticle (SiNP-RAFT)

SiNP-RAFT was synthesized according to our previously reported method[2] with some modifications as follows. At first, silica nanoparticles (SiNP) were prepared following a modified Stöber method. 25% Ammonia solution (7.6 mL), 99.9% pure ethanol (50 mL) and milli-Q water (76.5 mL) were mixed in a round bottom flask and stirred at 600 rpm. Subsequently, a mixture of TEOS (11.4 mL) and ethanol (114 mL) was added. The reaction mixture turned turbid white as SiO_2 nanoparticles formed after ~10 min. The reaction was allowed to continue for 8 h at room temperature. Thereafter, the particles were collected by centrifugation (10 000 rpm, 10 min) and washed by repeated redispersion in ethanol at least three times. Finally, the product was dried under vacuum at room temperature overnight.

Then, a suspension of SiO_2 nanoparticles (SINP) (14 g) in anhydrous toluene (200 mL) was added to a three-necked round-bottom flask by magnetic stirring under nitrogen. Based on the theoretical number of silanol groups (8 µmol/m2), an excess of APTES (2.37 g, 10.7 mmol) was then added and the mixture refluxed for 12 h at 130 °C under nitrogen. The mixture was then cooled to room temperature. Thereafter, the particles were collected by centrifugation (10000 rpm, 10 min) and washed with methanol at

least three times. Finally, the amino functionalized silica nanoparticles (SINP-NH2) was dried under vacuum at room temperature for two days. Finally, a solution of CPDB (0.385 g, 1.38 mmol), ethylchloroformate (132 µL, 1.38 mmol) and TEA (192 µL, 1.38 mmol) in THF (50 mL) was added to a three-necked round bottom flask (250 mL). The solution was purged with nitrogen and cooled in an ethanol-liquid nitrogen bath for 40 minutes at -70 °C. The stock solution of SiNP-NH_2 (7.0 g in 70 mL THF) was then added at -10 °C and the reaction allowed to proceed overnight at room temperature. The RAFT reagent modified silica nanoparticles (SiNP-RAFT) were collected by centrifugation (10 000 rpm, 10 min), washed with ethanol at least five times and dried under vacuum at room temperature.

1.3. References

1) Wan, W., Biyikal, M., Wagner, R., Sellergren, B. & Rurack, K. Fluorescent Sensory Microparticles that "Light-up" Consisting of a Silica Core and a Molecularly Imprinted Polymer (MIP) Shell. *Angewandte Chemie International Edition* **52**, 7023-7027, doi:10.1002/anie.201300322 (2013).

2) Shinde, S. *et al.* Sialic acid imprinted fluorescent core-shell particles for selective labeling of cell surface glycans. *Journal of the American Chemical Society* **137**, 13908-13912, doi:10.1021/jacs.5b08482 (2015).

2. Figures and tables

Table S1. Results from the characterization of core-shell particles.

		SiNP	SiNP-NH_2	SiNP-RAFT	P1	P_N1	P2
**FTIR**	I_{1728}/I_{1050} [a]	/	/	0	0.043	0.048	0.063
**TGA**	Weight loss excluding water, %	9.7	10.4	12.6	14.9	14.9	15.3
**DLS**	Average size, nm	266±15	322±7	295±3	407±4	410±25	443±65

a) Ratio of the intensity of the C=O (1728 cm^{-1}) over the Si-O-Si (1050 cm^{-1}) vibrations.

Table S2. Structure and properties of tested lipids

Abbreviated Name	Chemical structure	Mw	LogP	Charge
FP	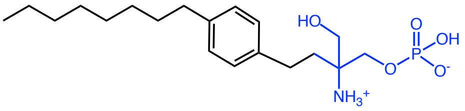	387	4.73	0
S1P	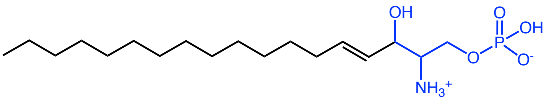	379	5.24	0
DPPA (Na)	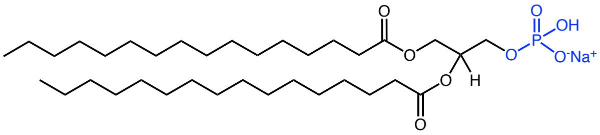	671	11.71	-1
AMP	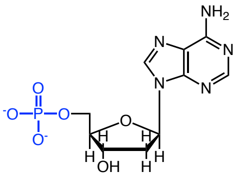	347	-3.1	-1
POPE	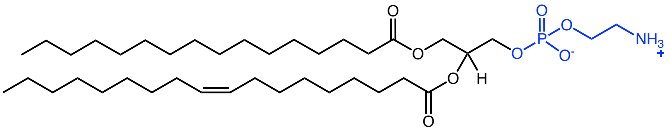	718	14.25	0
DPPC	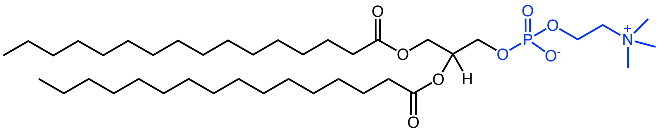	734	10.88	0
POPC	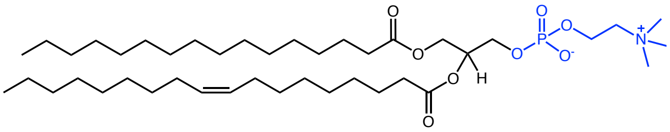	760	11.42	0

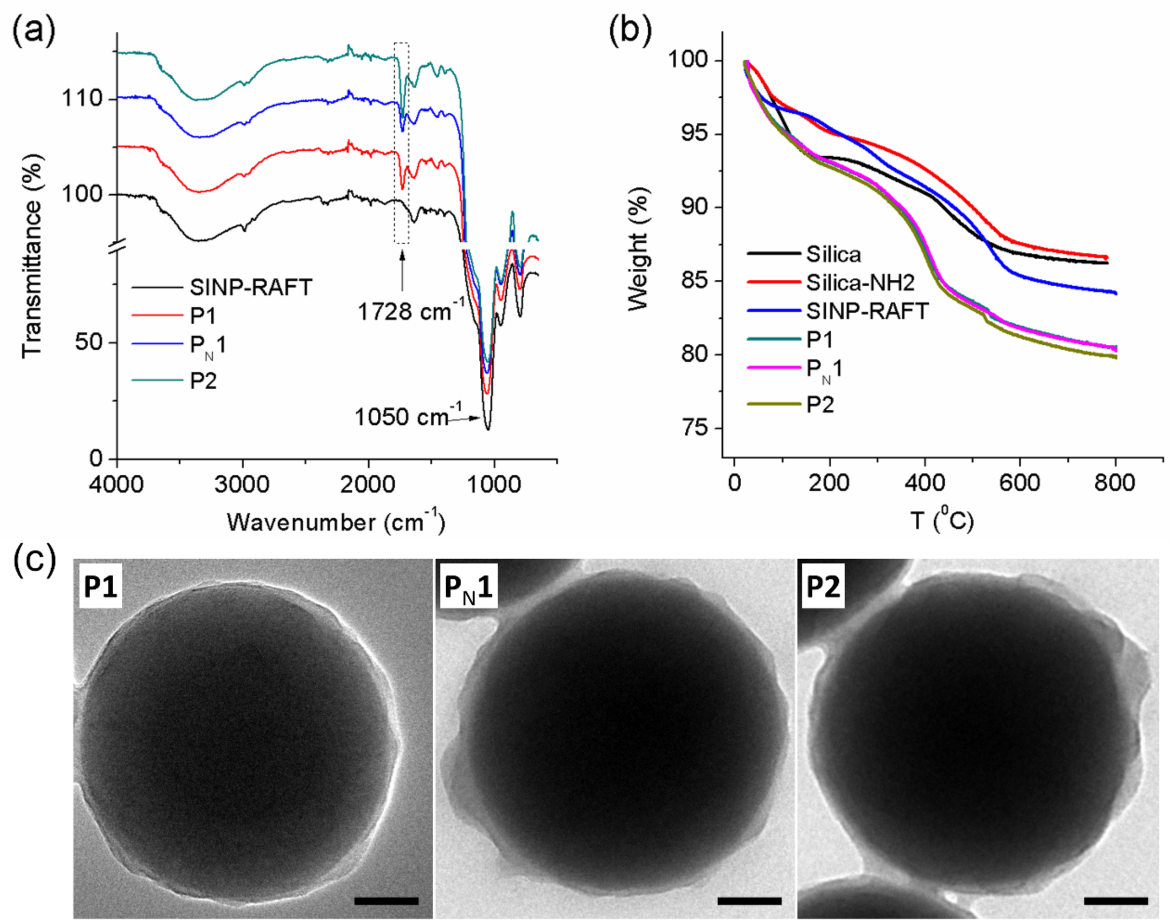

Figure S1. IR (a), TGA (b) and TEM (c) characterization results corresponding to the core-shell particles. Silica: silica nanoparticle; Silica-NH_2: APTES functionalized silica nanoparticle; SiNP-RAFT: RAFT functionalized Silica-NH2. Scale bar = 50 nm.

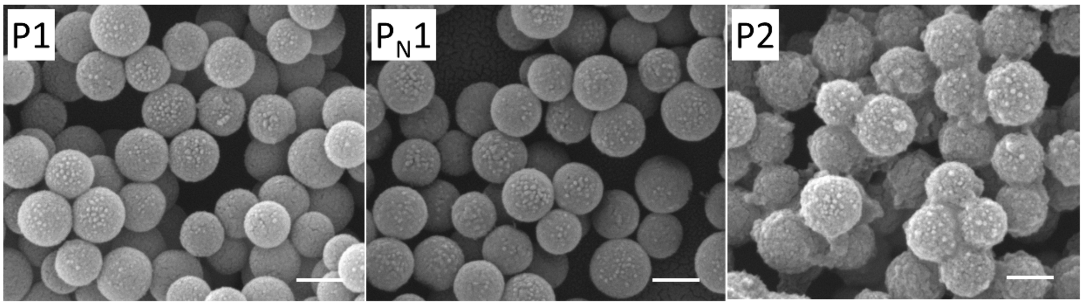

Figure S2. SEM images of P1, P$_N$1 and P2. Scale bar=200 nm.

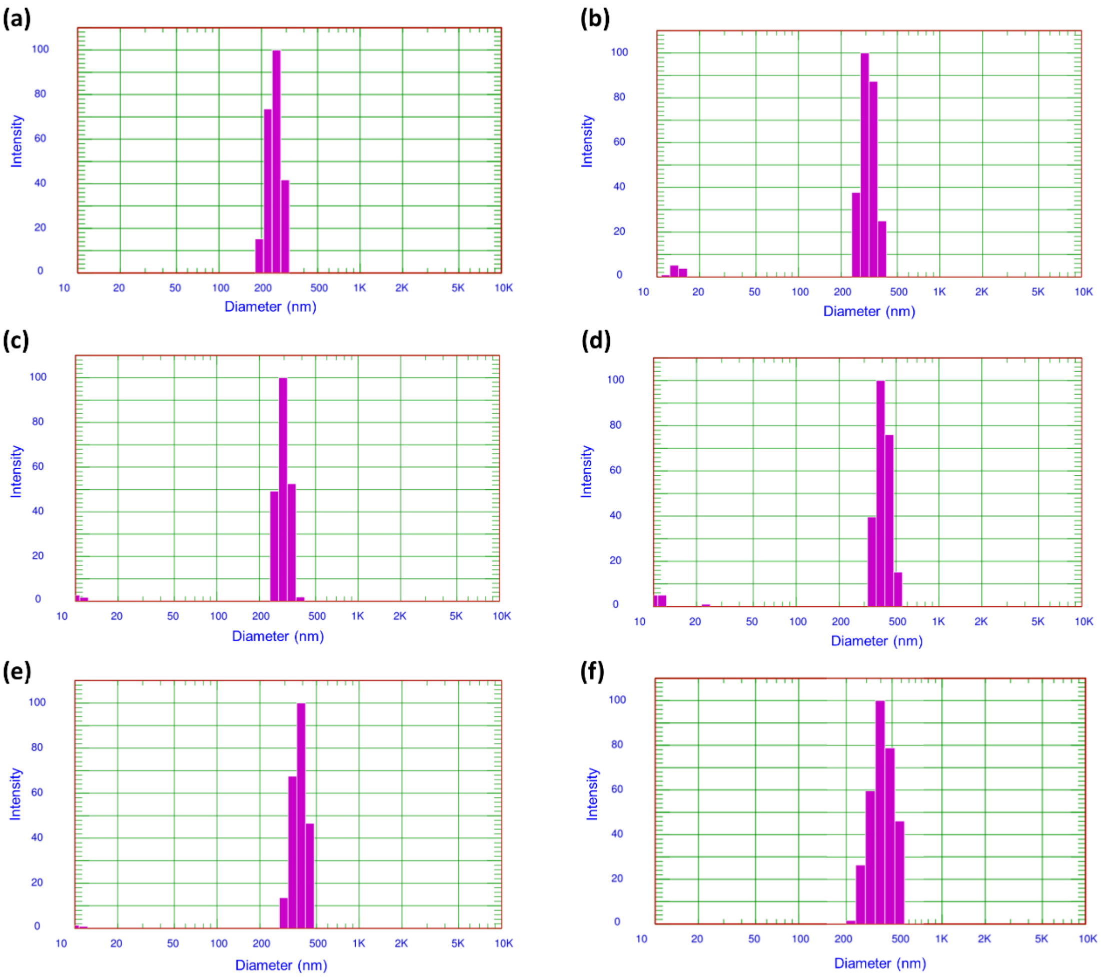

Figure S3. Particle size distribution of SiNP (a), SiNP-NH_2 (b), SiNP-RAFT (c), FP-imprinted nanoparticle (P1, d), non-imprinted nanoparticle (P_N1, e) and DPPA-imprinted nanoparticle (P2, f), measured by dynamic light scattering (DLS) in methanol.

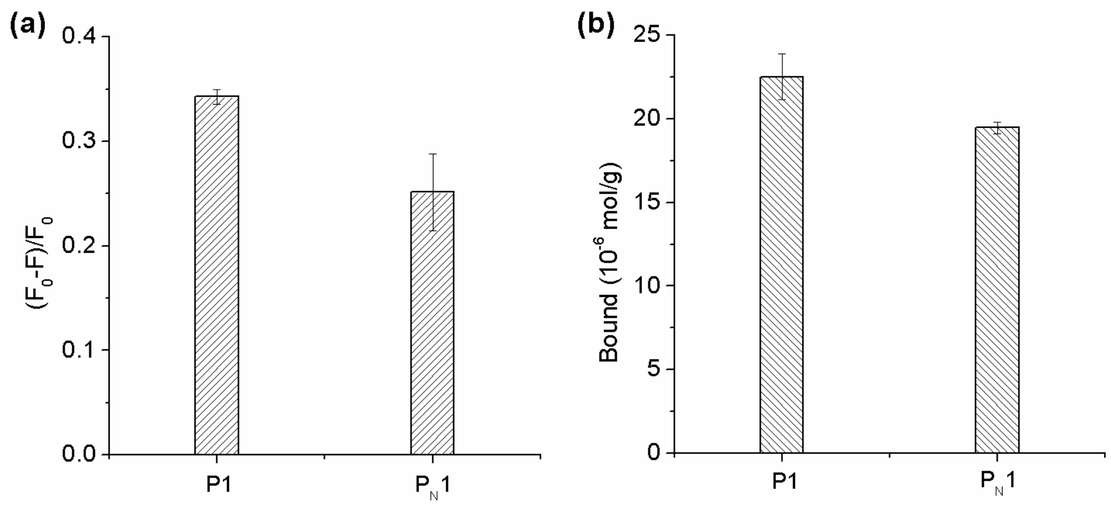

Figure S4. Fluorescence response (a) and adsorbed amount (b) after incubation of P1 and P_N1 with the template FP(TBA) (100 µM) in methanol/water=95/5. Polymer concentration: 1 mg/mL.

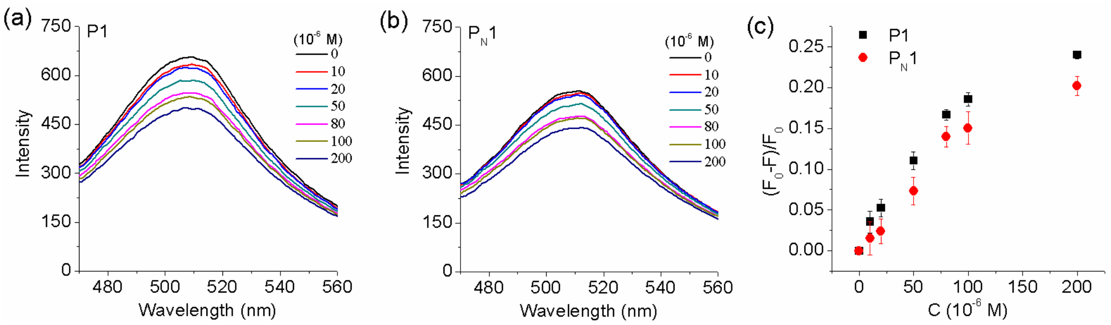

Figure S5. Fluorescence spectra of P1 (a) and P_N1 (b) in presence of FP(TBA) in methanol/water (95/5) (λ_{ex}= 440 nm), and corresponding dose-response curves (c) for the fluorescence emission intensity at 512 nm. Polymer concentration: 2.5 mg/mL.

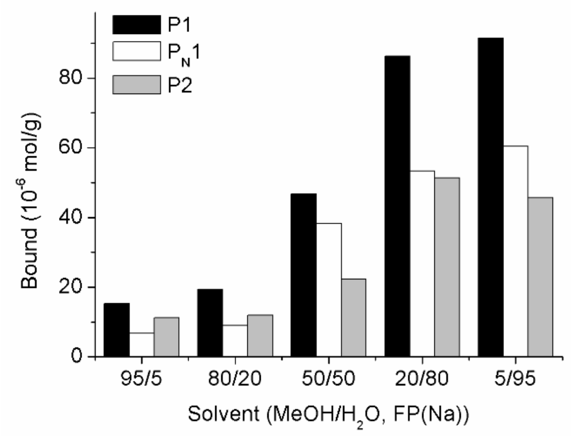

Figure S6. Adsorbed amount of FP(Na) to the core-shell particles in different methanol/water (MeOH/H_2O) solvent systems. Test molecule concentration: 100 µM; polymer concentration: 1 mg/mL.

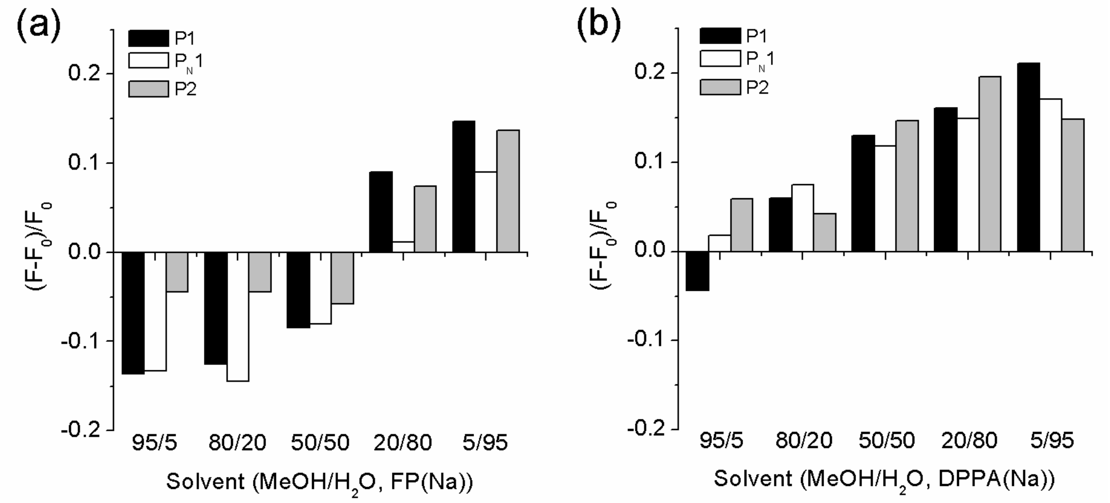

Figure S7. Fluorescence response of the core-shell particles after incubation with solutions of FP(Na) and DPPA(Na) in different methanol/water (MeOH/H_2O) solvent systems. Test molecule concentration: 100 µM; polymer concentration: 1 mg/mL.

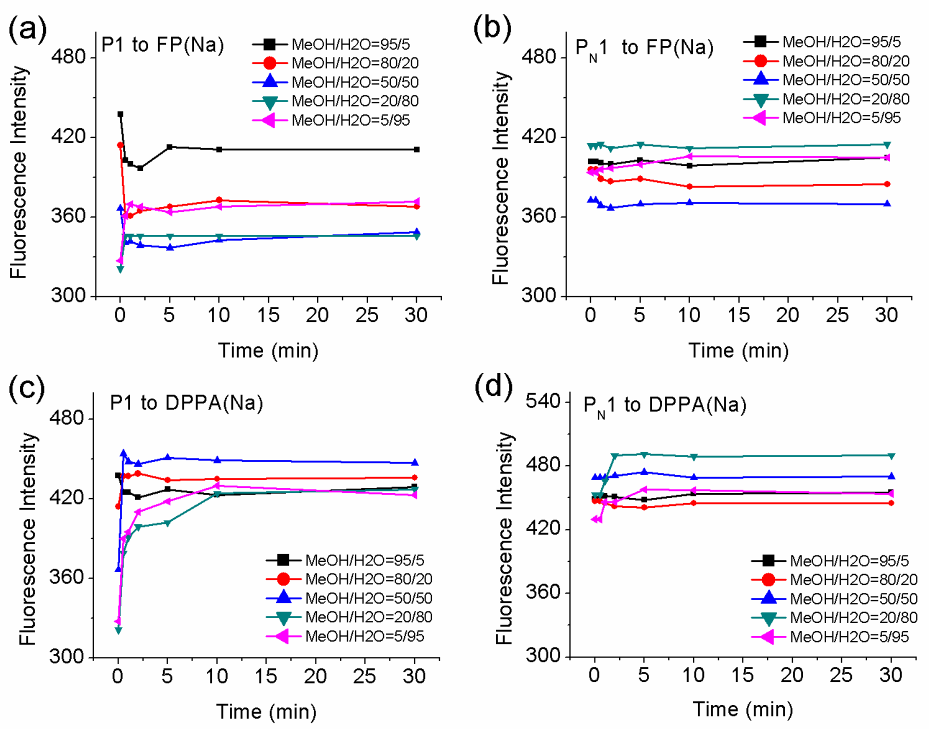

Figure S8. Fluorescence emission intensity versus time for the indicated core/shell polymers after addition of lipids in methanol/water of different volume ratios. Polymer concentration: 1 mg/mL; test molecule concentration: 100 µM.

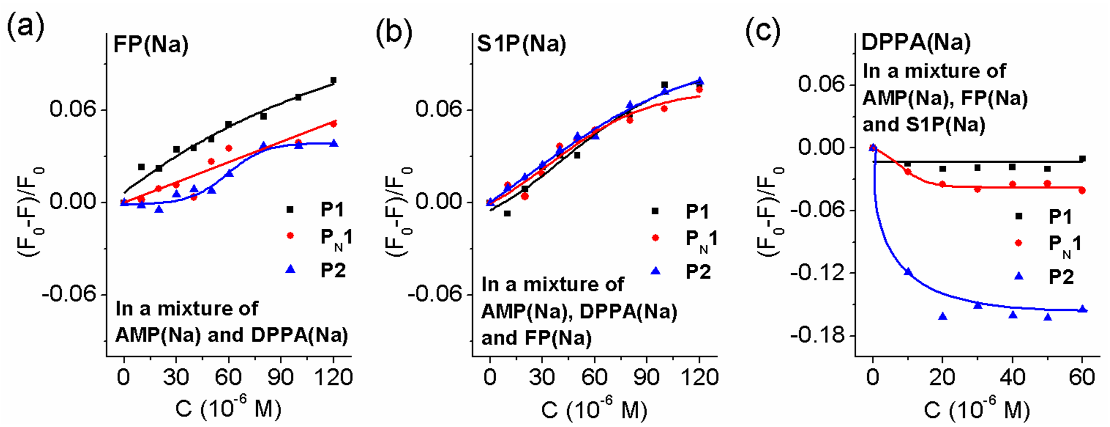

Figure S9. Effect of interferences on fluorescence response of polymers (P1, P_N1 and P2) to FP(Na) (a), S1P(Na) (b) and DPPA(Na) (c), respectively. Polymer concentration: 1 mg/mL; solvent: methanol/water=1/1 (v/v). (a) The concentration of AMP(Na) and DPPA(Na) were both set at 100 µM; (b) the concentration of AMP(Na) and DPPA(Na) were both set at 100 µM, and the concentration of FP(Na) was set at 120 µM; (c) the concentration of AMP(Na), FP(Na) and S1P(Na) were all set at 100 µM. The lines are drawn as a guide for the eye.

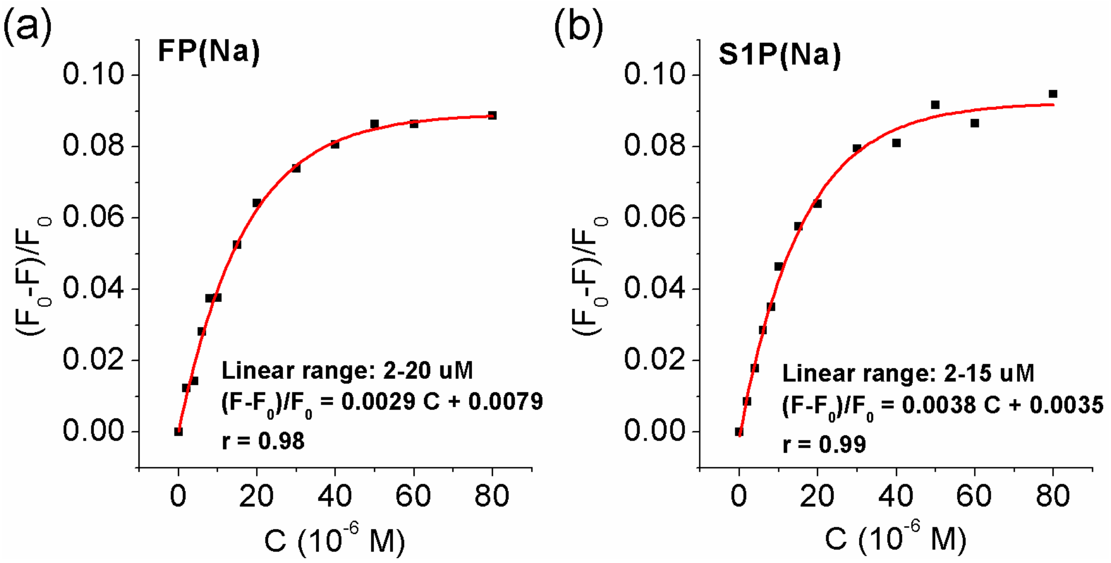

Figure S10. Dose-response of P1 to the targets of FP(Na) (a) and S1P(Na) (b) in diluted human serum (4-times dilution). Polymer concentration: 1mg/mL.
